# Comparison between melatonin versus melatonin and photobiomodulation versus photobiomodulation in the treatment of Alopecia X in German Spitz dogs: Clinical, randomized, double-blind, parallel, non-inferiority protocol

**DOI:** 10.1371/journal.pone.0304605

**Published:** 2024-06-11

**Authors:** Flaviana Amado Martins, Gabriel Almeida da Silva, Ana Paula Ligeiro de Oliveira, Cinthya Cosme Gutierrez Duran, Ovidiu Constantin Baltatu, Rodrigo Labat Marcos, Anna Carolina Ratto Tempestini Horliana, Stella Regina Zamuner, Jose Antônio Silva Júnior

**Affiliations:** 1 Biophotonics Medicine, UNINOVE, Sao Paulo, Sao Paulo, Brazil; 2 Medicine, UNINOVE, Sao Paulo, Sao Paulo, Brazil; 3 College of Medicine and Health Sciences, Khalifa University of Science and Technology, Abu Dhabi, United Arab Emirates; Massachusetts General Hospital, UNITED STATES

## Abstract

Canine Alopecia X is a non-inflammatory hair loss disorder of unknown etiology that predominantly affects German Spitz dogs. Treatment modalities include hormone and/or melatonin supplementation and low trauma microneedling. Melatonin influences hair growth and pigmentation in several species and presents a low risk of adverse effects when used in dogs with Alopecia X. Photobiomodulation (PBM) is frequently used in human androgenetic alopecia and alopecia areata; despite this, PBM remains unexplored in canine Alopecia X. To address this knowledge gap, sixty dogs of both sexes will be randomly assigned to three groups: (i) melatonin only group (3 mg/Kg, n = 20); (ii) PBM only group (diode laser, wavelength 660nm, 100mw power, with 3 J/point, 2 sessions/week for 3 months, n = 20); (ii) PBM + melatonin group (n = 20). The objective is to determine the potential of PBM alone or in conjunction with melatonin supplementation in promoting hair regrowth (hair density and diameter) by means of dermatoscopy and planimetry over a period of 90 days.

## Introduction

Alopecia X is a non-inflammatory hair loss disorder of plush coated dogs that mainly occurs in male German and Italian Spitz breeds. In Spitz breed, the disease accounts for nearly 23% of all non-inflammatory hair loss disorders [1]. Alopecia X is a poorly understood skin disorder featuring symmetrical truncal, cervical, perineal, and/or caudal aspects of the hindlimbs, hair loss, and/or skin hyperpigmentation with no pruritus or systemic signs [2]. Treatments for Alopecia X are variable and include orchiectomy or ovariohysterectomy, microneedling, methyltestosterone and growth hormone supplementation, trilostane, mitotane, deslorelin and/or melatonin [3, 4]. Melatonin, a naturally occurring hormone produced by the pineal gland in response to darkness, has been identified as a modulator of hair growth and pigmentation across species [3, 4]. Furthermore, melatonin supplementation stands out as a secure, economical, and efficacious standalone treatment for dogs experiencing alopecia, including alopecia X [5, 6]. Research findings demonstrate that melatonin is capable of stimulate hair regrowth in dogs with Alopecia X, underscoring its efficacy as an independent treatment [7, 8].

In the late 1960s, Endre Mester conducted experiments using low-power lasers that stimulated hair regrowth in rats. This discovery marked the inception of photo biostimulation through Low-Level Laser Therapy (LLLT) or Photobiomodulation (PBM) [9]. More recently, some authors have revealed that PBM can promote an increase in anagen follicles and stimulates mitochondria in the stem cells of the follicular bulge in human alopecic disorders [10]. As far as we know, no attempts were made using PBM for Alopecia X treatment. Alternative treatment modalities can reduce the use of drugs as recommended by some authors [11].

Given the limited number of studies focused in PBM and non-inflammatory alopecias, including Alopecia X, additional studies are needed to verify PBM as a potential treatment choice and also establish best therapeutic laser devices and optimal dosage parameters for specific skin conditions. Large-scale, randomized, controlled, and double-blinded *in vivo* studies are necessary [12]. A systematic review of PBM in Veterinary Medicine underscores the scarcity of high-quality evidence regarding the clinical effectiveness of laser and LED therapy in horses, dogs, and cats [13]. A study examining the impact of PBM on dogs with non-inflammatory alopecias, encompassing diverse breeds, sexes, and ages [14], demonstrated favorable outcomes regarding hair growth and coat quality. However, given the limited sample size of seven animals, the authors concluded that additional studies are needed.

The main hypothesis of our study is that PBM alone or combined with melatonin can cause hair regrowth in German Spitz dogs with Alopecia X.

## Materials and methods

### Study design

**Fig 1:**
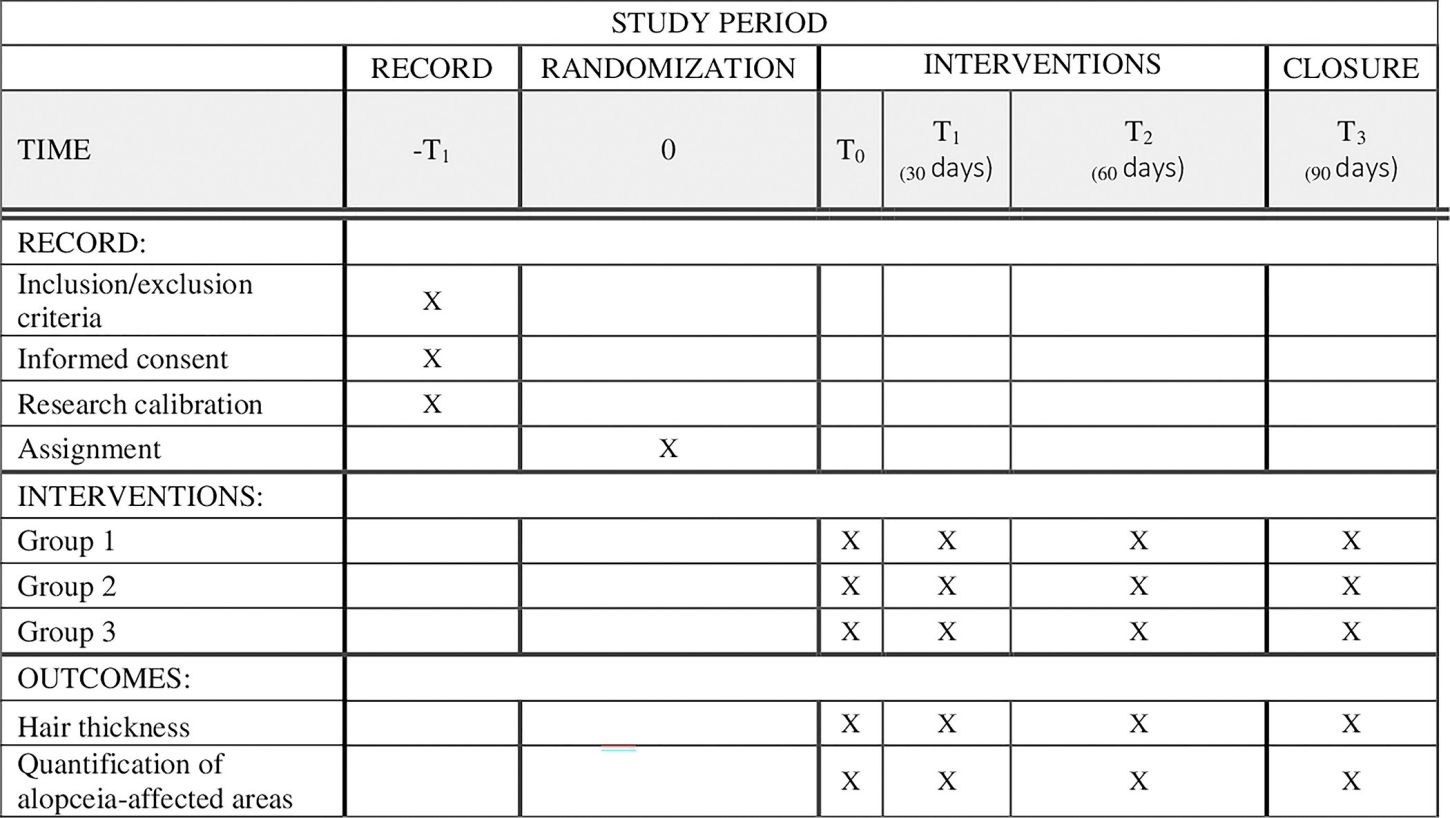
SPIRIT flow diagram of the study.

Randomized, double-blind, parallel, non-inferiority clinical protocol. This study protocol was deposited in animalstudyregistry.org (DOI 10.17590/asr.0000344). The project received approval from the Research Ethics Committee of Nine of July University, process: 8930131223. The study will start on May 6^th^, 2024, and finish on March 28th, 2025. All owners should provide a signed informed consent form approved by the Ethics Committee to apply to the study. Future methodological changes deemed necessary for the completion of the study will be promptly communicated to the CEP and disclosed in future publications. All owners will have access to the dog’s clinical records during and after the study’s conclusion. The SPIRIT flow diagram of the study is presented in Fig 1.

### Description of the sample

**Fig 2:**
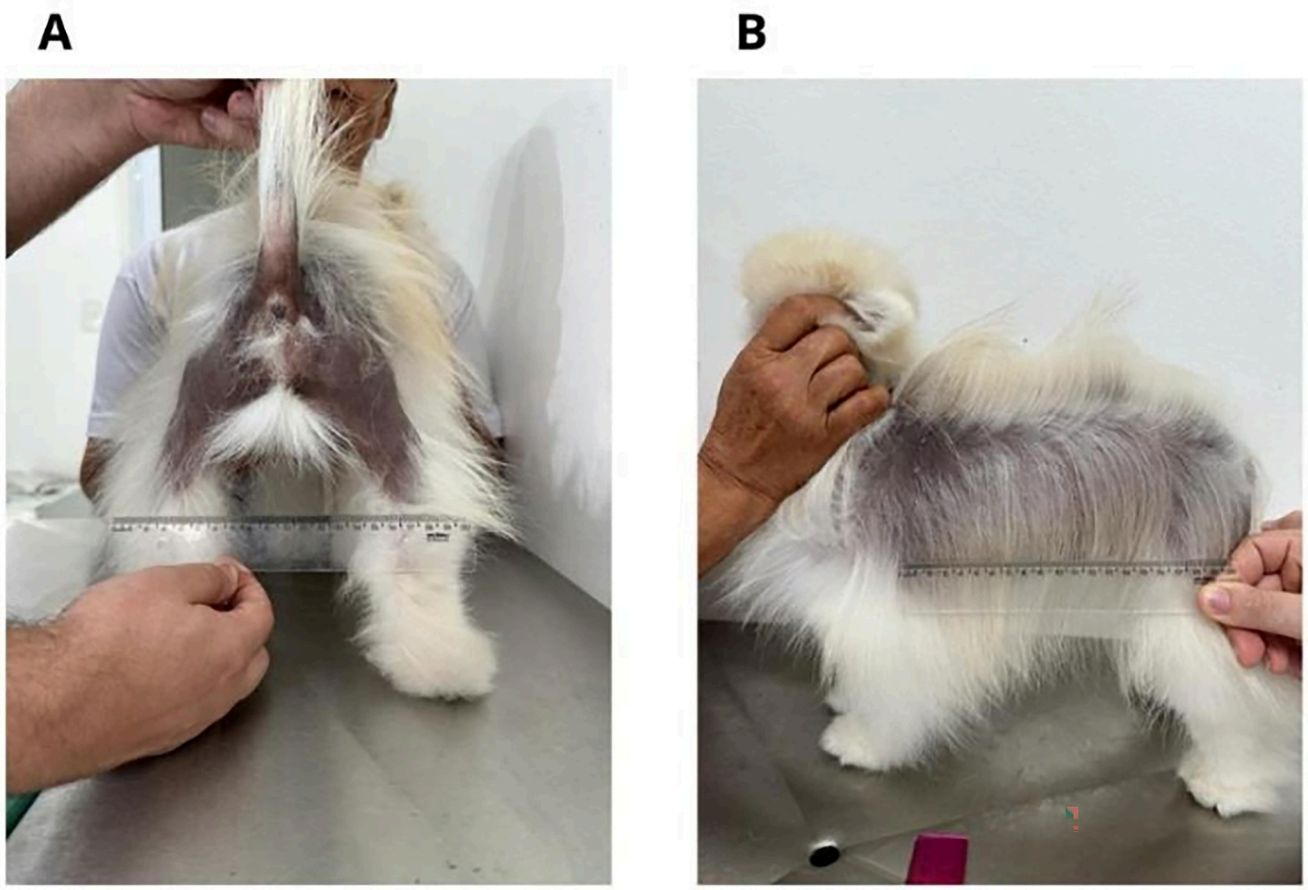
Fig 2 represents caudal (2A) and lateral (2B) areas affected by Alopecia X in the same dog.

Client-owned Spitz dogs affected by Alopecia X (Fig 2) will be enrolled for the study at Dermacare Vet—Veterinary Dermatology and Photobiomodulation Center in São Paulo, SP, Brazil.

#### Inclusion/exclusion criteria

Dogs will be included in this study based on the following criteria:

- German Spitz with Alopecia X;
- male and female;
- neutered for at least 6 months before study enrollment;
- onset of hair loss between 1 to 6 year-old;
- 1 to 10 Kg weight;
- truncal and/or cervical and/or perineal hair loss with or w/o wooly coat quality with or w/o hyperpigmentation;
- no pruritus or systemic disease;
- no bacterial pyoderma;
- hematological, thyroid (FT4 by equilibrium dialysis + TSH) and adrenal tests (dexamethasone suppression test) at normal reference range;
- no previous topical or systemic corticosteroidal treatment for at least 30 days before study enrollment;
- prior treatment with Simparic^®^ for at least 30 days before study enrollment.

The diagnosis of alopecia X will be based on anamnesis, physical and dermatological examination, dermatoscopy, and exclusion of endocrine disorders.

Dogs will be excluded from the study based on the following criteria:

- Dogs positive for *Malassezia* spp and/or cocci in cytological examination;
- unresponsive to standard antifungal and antibacterial clinical treatment;
- with prutitus and/or previously treated with melatonin for at least 30 days before study enrollment.

#### Sample size calculation

Sample size was calculated by McNemar’s test. The minimum number of dogs is fifty-six (n = 56). In order to standardize the total number of dogs per group, we decide to allocate twenty dogs in each one.

### Training of examiners

All examiners have experience in Veterinary Dermatology, PBM, and data analysis.

### Randomization

All dogs (n = 60) will be randomly assigned in three groups using a random sequence generator program available at https://www.sealedenvelope.com/.

### Evaluation before and after treatment

#### Planimetry

Hair loss and regrowth will be evaluated by digital dotmatrix planimetry. High quality images from four anatomical projections (caudal, dorsal, right lateral and left lateral) will be acquired using an iPhone 14 Pro Max^®^ at a distance of 50 cm from each dog. All illuminations parameters will be similar for all dogs. In order to set a standard measuring scale in cm a ruler will be hold next to dogs in all photos. Digital planimetry will be done in Image J^®^ (freeware) before and after the treatments as follows: menu>analyze>set scale (parameters: pixel aspect ratio = 1.0; unit of length = cm; global). After that, the digital dotmatrix will be plotted over all images (plugins>analyze>grid). The difference between hair density before and after treatments will be recorded as the percentage of hair regrowth and classified as excellent regrowth = ER (80–100%); good regrowth = BR (60–79%); reasonable regrowth = RR (40–59%); and minimum regrowth = MR (0–39%) [15]. Areas of maximum alopecia will be selected to assessment of hair regrowth.

#### Dermatoscopy

**Fig 3:**
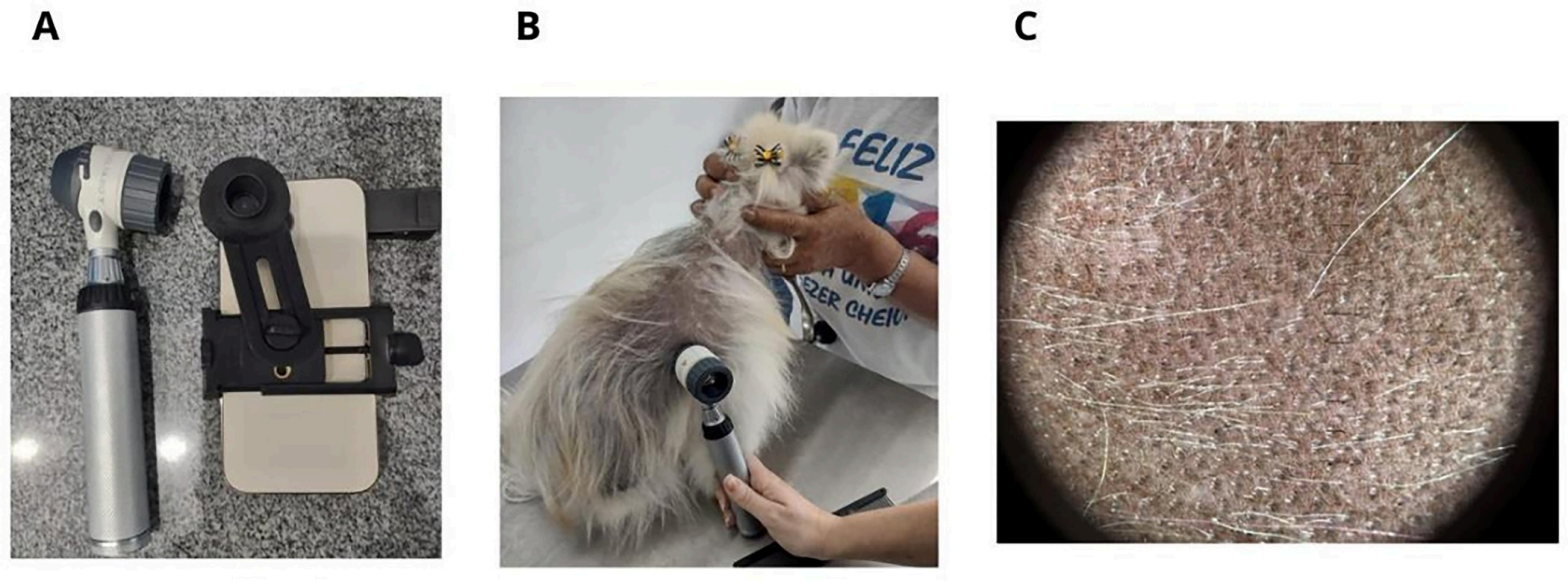
Dermatoscopic device (3A), procedure (3B) and features in a dog with alopecia X (3C).

Hair thickness (in mm) and hair density (total number of hairs per mm^2^), will be evaluated before and after treatments using a Heine Delta 20T^®^ device (Fig 3A). All photos will be acquired using an iPhone 14 Pro Max^®^ with a 2X zoom. All images will be analyzed in Image J^®^ as follows: menu>analyze>set scale (parameters: pixel aspect ratio = 1.0; unit of length = mm; global).

### Experimental groups

**Fig 4:**
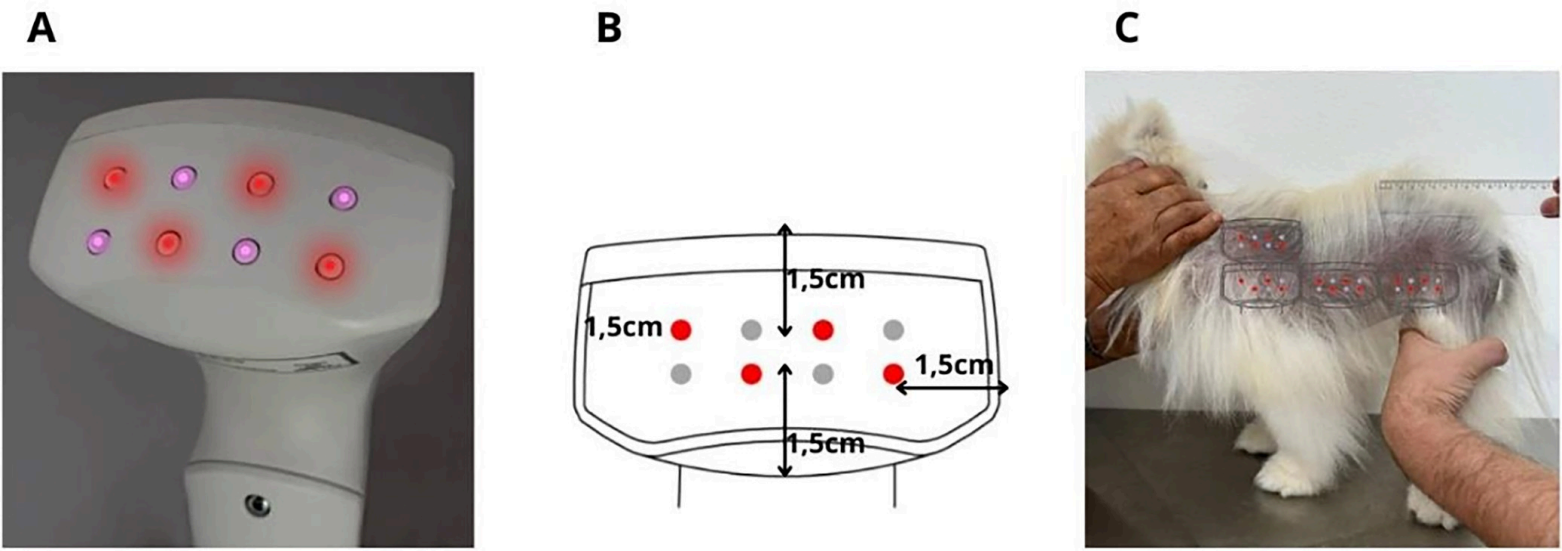
Laser device displaying all diodes online (4A); red light arrangement and distance between diodes and device boundaries (4B), and device application points in areas with alopecia (4C).

Sixty dogs (n = 60) will be randomly assigned in three different groups: (i) melatonin + PBM simulation (n = 20) where melatonin will be administered orally at 3 mg/Kg every 12 hours for 3 months. PBM simulation will be done with a non-laser light emitting device; (ii) PBM only (n = 20) where photobiomodulation will be performed twice a week (72-hour interval), delivering 3J/point (radiant exposure at target = 169J/cm^2^) of red light covering all areas of hair loss (Fig 4C) for 90 days. For this end, an e-light IRL^®^ device (Fig 4A), equipped with 4 diodes with a wavelength of 660nm (red, 100mW per diode) and 4 diodes with a 808nm (infrared, 120 mW per diode) (Fig 4B). During the procedure, the four infrared laser diodes will remain offline and thus a total power of 400mW from red laser will be delivered.; (iii) PBM + melatonin (n = 20) where melatonin will be administered orally at 3 mg/kg every 12 hours. Simultaneously, photobiomodulation will be performed twice a week (72-hour interval), delivering 3J/point (radiant exposure at target = 169J/cm^2^) of red light covering all areas of hair loss (Fig 4C) for 90 days. For this end, an e-light IRL^®^ device (Fig 4A), equipped with 4 diodes with a wavelength of 660nm (red, 100mW per diode) and 4 diodes with a 808nm (infrared, 120 mW per diode) (Fig 4B). During the procedure, the four infrared laser diodes will remain offline and thus a total power of 400mW from red laser will be delivered. All PBM parameters are shown in Table 1.

**Table 1:** Describes device features and variables.

Parameters	
Peak wavelength [nm]	660nm
Spectral bandwidth (FWHM) [nm]	2
Operating mode	Continuous wave
Average radiante power [mW]	100
Polarization	Linearly polarized
Irradiance at aperture [mW/cm^2^]	5650
Beam profile	Multimode
Beam spot size at target [cm^2^]	0,0177
Exposure duration per site [s]	30
Radiant exposure at target [J/cm^2^]	169
Radiant energy per site [J]	3
Application technique	contact with the skin surface, each application point is 1.5 cm away from the other
Frequency of treatment sessions	2x per week, during 13 weeks
Number of treatment sessions	26

FWHM, full width at half maximum; J, Joule.

### Blinding

The principal researcher is the only one aware of all treatments pursued in each dog. Other research team members will be unaware (blinded).

The research team will consist of the following roles:

- Veterinarian: performs the clinical assessment of all candidate dogs for the study using inclusion and exclusion criteria. Blinded to the groups, data acquisition, image analysis, statistical results.
- Image examiner: performs image analysis related to dermatoscopy and planimetry. Blinded to the groups, data acquisition, statistical results.
- PBM practitioner: performs PBM in all enrolled dogs. Blinded to the groups, data acquisition, image analysis, statistical results.
- Statistician: performs data analysis, blinded to the groups, data acquisition, image analysis.
- Owners: blinded to the groups, data acquisition, image analysis, statistical results.

### Outcomes

The evaluated outcomes will be hair density measured by planimetry and dermatoscopy; and hair diameter as previously described.

### Discontinuation criteria

- Dogs with adverse drug reactions to melatonin;
- Non-attendance to scheduled treatment and evaluations;
- Dogs undergoing additional treatment prescribed by other veterinarian and/or performed by owner

### Strategies to enhance adherence

○ Continue treatment beyond the 90 days study period.

### Trial timeline

**Fig 5:**
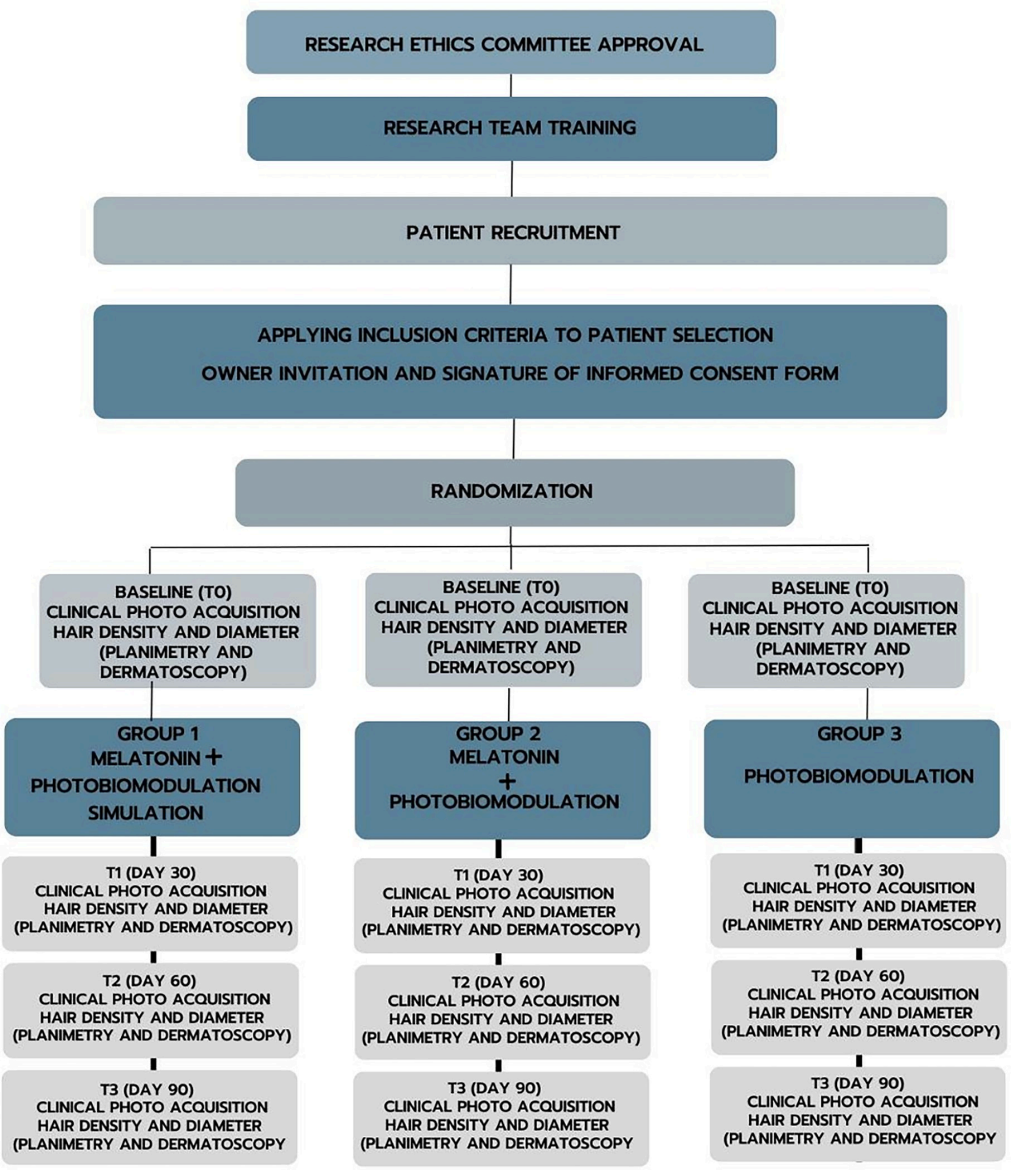
Study design of the study “Comparison between melatonin versus melatonin and photobiomodulation versus photobiomodulation in the treatment of Alopecia X in German Spitz dogs: Clinical, randomized, double-blind, parallel, non-inferiority protocol”.

Hair regrowth assessment will be evaluated at T1, T2, and T3 (T0 = day 0, T1 = 30 days; T2 = 60 days, and T3 = 90 days). The study design is depicted in Fig 5.

### Statistical analysis

Data related to hair regrowth before and after the treatments will be tested for normality by Shapiro Wilk and Kolmogorov Smirnov tests. ANOVA test followed by Tukey Multiple Comparison test will be used for normal distribution data. If data display non-normal distribution, the analysis will be conducted using the same design but adjusted for a gamma distribution, followed by the Wald Multiple. *P*<0.05 will be considered significant. Statistical analysis will be performed with commercial software SAS 9.4.

## Discussion

Simple, reliable, and safe new treatment modalities for canine Alopecia X are urgently needed. Aside cosmetic issues, dogs with alopecia X are prone to develop secondary bacterial pyoderma, fungal infections, solar damage, and thermal stress. Our study aims to investigate the efficacy and safety of PBM alone or combined with melatonin in dogs with Alopecia X. We hypothesize that PBM alone increases hair density and diameter, promoting hair regrowth. Additionally, PBM will be tested as a single treatment to avoid medication for this condition. Nonetheless, both interventions were well-tolerated, with minimal side effects observed.

Optimal parameters for therapeutic lasers and dosage regimens for specific skin conditions should be determined and standardized in large scale studies, including different species, breeds, sexes, and ages [12, 14]. Until now, very few studies have addressed these issues. In addition, no standardized protocols for Alopecia X have been published until now which makes this protocol an innovation in the field.

Due to the lack of evidence based scientific data regarding PBM effects on canine Alopecia X, it can be difficult for owners to fully adhere to the pre-established protocols described in this study. This can potentially decrease the total number of dogs enrolled in the study. An extensive call-to-action through advertisements on social media, pet shops, kennel clubs, dermatology, and diagnostic centers will be carried out to reach the maximum number of patients with Alopecia X. Despite limitations, our study offers valuable preliminary data regarding the safety and efficacy of PBM in treating Alopecia X in Spitz dogs.

## Supporting information

S1 Checklist SPIRIT 2013 Checklist of the study “Comparison between melatonin versus melatonin and photobiomodulation versus photobiomodulation in the treatment of Alopecia X in German Spitz dogs: Clinical, randomized, double-blind, parallel, non-inferiority protocol”. (DOC)
