# Correction: Comparison between melatonin versus melatonin and photobiomodulation versus photobiomodulation in the treatment of Alopecia X in German Spitz dogs: Clinical, randomized, double-blind, parallel, non-inferiority protocol

**DOI:** 10.1371/journal.pone.0320388

**Published:** 2025-03-10

**Authors:** Flaviana Amado Martins, Gabriel Almeida da Silva, Ana Paula Ligeiro de Oliveira, Cinthya Cosme Gutierrez Duran, Ovidiu Constantin Baltatu, Rodrigo Labat Marcos, Anna Carolina Ratto Tempestini Horliana, Stella Regina Zamuner, Jose Antônio Silva Júnior

In the Abstract section, there is an error in the fifth sentence. The correct sentence is: To address this knowledge gap, sixty dogs of both sexes will be randomly assigned to three groups: (i) melatonin only group (3 mg/dog, n = 20); (ii) PBM only group (diode laser, wavelength 660nm, 100mw power, with 3 J/point, 2 sessions/week for 3 months, n = 20); (ii) PBM + melatonin group (n = 20).

In the Experimental groups subsection of Materials and Methods, there is an error in the first and fourth sentences. The first correct sentence is: Sixty dogs (n =  60) will be randomly assigned in three different groups: (i) melatonin +  PBM simulation (n =  20) where melatonin will be administered orally at 3 mg/dog every 12 hours for 3 months. The fourth correct sentence is: During the procedure, the four infrared laser diodes will remain offline and thus a total power of 400mW from red laser will be delivered.; (iii) PBM +  melatonin (n =  20) where melatonin will be administered orally at 3 mg/dog every 12 hours.

## References

[1] Amado Martins F, Almeida da Silva G, Ligeiro de Oliveira AP, Gutierrez Duran CC, Constantin Baltatu O, Labat Marcos R, et al. Comparison between melatonin versus melatonin and photobiomodulation versus photobiomodulation in the treatment of Alopecia X in German Spitz dogs: Clinical, randomized, double-blind, parallel, non-inferiority protocol. PLoS One. 2024;19(6):e0304605. doi: 10.1371/journal.pone.0304605 38861499 PMC11166340

